# Application of a simplified definition of diastolic function in severe sepsis and septic shock

**DOI:** 10.1186/s13054-016-1421-3

**Published:** 2016-08-04

**Authors:** Michael J. Lanspa, Andrea R. Gutsche, Emily L. Wilson, Troy D. Olsen, Eliotte L. Hirshberg, Daniel B. Knox, Samuel M. Brown, Colin K. Grissom

**Affiliations:** 1 Critical Care Echocardiography Service, Intermountain Medical Center, 5121 South Cottonwood Street, Murray, UT 84157 USA; 2 Division of Pulmonary and Critical Care Medicine, University of Utah, 30 North 1900 East, 701 Wintrobe Building, Salt Lake City, UT 84132 USA; 3 Department of Anesthesiology, University of Utah, 30 North 1900 East, 701 Wintrobe, Salt Lake City, UT 84132 USA; 4 Division of Pediatric Critical Care, Department of Pediatrics, University of Utah, 295 Chipeta Way, Salt Lake City, UT 84108 USA; 5 Division of Pulmonary, Allergy, and Critical Care Medicine, University of Massachusetts, 55 Lake Avenue North, Worchester, MA 01655 USA

**Keywords:** Diastolic, Sepsis, Echocardiography, Classification, Shock

## Abstract

**Background:**

Left ventricular diastolic dysfunction is common in patients with severe sepsis or septic shock, but the best approach to categorization is unknown. We assessed the association of common measures of diastolic function with clinical outcomes and tested the utility of a simplified definition of diastolic dysfunction against the American Society of Echocardiography (ASE) 2009 definition.

**Methods:**

In this prospective observational study, patients with severe sepsis or septic shock underwent transthoracic echocardiography within 24 h of onset of sepsis (median 4.3 h). We measured echocardiographic parameters of diastolic function and used random forest analysis to assess their association with clinical outcomes (28-day mortality and ICU-free days to day 28) and thereby suggest a simplified definition. We then compared patients categorized by the ASE 2009 definition and our simplified definition.

**Results:**

We studied 167 patients. The ASE 2009 definition categorized only 35 % of patients. Random forest analysis demonstrated that the left atrial volume index and deceleration time, central to the ASE 2009 definition, were not associated with clinical outcomes. Our simplified definition used only e′ and E/e′, omitting the other measurements. The simplified definition categorized 87 % of patients. Patients categorized by either ASE 2009 or our novel definition had similar clinical outcomes. In both definitions, worsened diastolic function was associated with increased prevalence of ischemic heart disease, diabetes, and hypertension.

**Conclusions:**

A novel, simplified definition of diastolic dysfunction categorized more patients with sepsis than ASE 2009 definition. Patients categorized according to the simplified definition did not differ from patients categorized according to the ASE 2009 definition in respect to clinical outcome or comorbidities.

**Electronic supplementary material:**

The online version of this article (doi:10.1186/s13054-016-1421-3) contains supplementary material, which is available to authorized users.

## Background

Diastolic dysfunction is associated with greater preceding fluid administration in the resuscitation of patients with sepsis, and is also associated with elevated left ventricular filling pressures [1–3]. Diastolic dysfunction is common in patients with severe sepsis or septic shock, but previous studies have used various definitions, reporting variable incidences (20–67 %) [1, 4–14]. Our group previously reported classification of diastolic dysfunction in 78 critically ill patients with severe sepsis or septic shock using three different definitions (Additional file 1: Table S1) [1]. The incidence of diastolic dysfunction ranged from 1.4 % to 59.4 %, depending on the definition employed, highlighting the need for a consistent, reproducible definition of diastolic dysfunction among patients with sepsis.

The American Society of Echocardiography (ASE 2009) guidelines categorize diastolic dysfunction using several echocardiographic measurements [15]. However, the ASE 2009 definition does not unambiguously categorize patients. Almost half of echocardiograms have measurements that are inconsistent, resulting in poor interreader agreement [16]. Some measurements are difficult or impossible to measure in the presence of tachycardia or atrial fibrillation, conditions common in the critically ill. The ratio of early diastolic velocity of mitral inflow to mitral annular velocity (E/e′) has a strong association with left atrial pressure [3] and is often measurable in critically ill patients. We hypothesized that a simplified definition using septal e′ and E/e′ might more consistently and unambiguously categorize diastolic function in patients with sepsis. We sought to determine whether a simplified approach to diastolic assessment is feasible in patients with severe sepsis and septic shock, and whether this simplified definition was associated with clinical outcomes different from those associated with the ASE 2009 definitions.

## Methods

### Study design

This prospective, observational study was conducted between October 2012 and December 2013 at Intermountain Medical Center, a 450-bed academic, tertiary care hospital, with patients admitted to the 24-bed shock trauma intensive care unit (ICU) or the 12-bed respiratory ICU. In these ICUs, transthoracic echocardiography (TTE) is routinely performed for patients with severe sepsis or septic shock at the time of ICU admission. The protocol was approved by the Intermountain Institutional Review Board with a waiver of informed consent.

### Patients

We screened patients between October 2012 and December 2013 admitted with severe sepsis or septic shock defined by the then-current 1992 American College of Chest Physicians/Society of Critical Care Medicine consensus criteria [17] and operationalized by recent large sepsis trials [18–20]. Patients met criteria for inclusion if they (1) were at least 18 years of age, (2) had a clinically suspected infection, (3) had two or more systemic inflammatory response syndrome criteria, and (4) had either septic shock (systolic blood pressure <90 mmHg despite an intravenous fluid challenge ≥20 ml/kg or infusion of any dose of vasopressor medications) or severe sepsis (defined in this study as serum lactate ≥4 mmol/L).

### Transthoracic echocardiography

TTE was performed using a Philips iE33 ultrasound system (Philips Medical Systems, Bothell, WA, USA). Patients were excluded if their TTE was performed more than 24 h after onset or if the image quality was so poor as to be uninterpretable. All TTE was performed by a cardiac sonographer. Studies were interpreted by the second author (ARG), reviewed and formatted by an advanced cardiac sonographer (TDO), followed by a consensus interpretation by two level II echocardiographers (CKG, MJL). All readers were blinded to clinical outcomes.

**Fig. 1:**
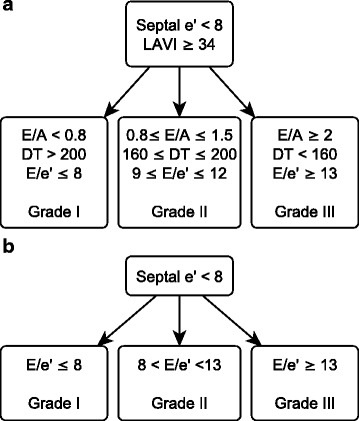
**a** American Society of Echocardiography (ASE 2009) definition of diastolic dysfunction [15]. The difference in duration flow between atrial reversal of flow and atrial inflow in late diastole duration and change in early diastolic velocity of mitral inflow (E) to late diastolic velocity of mitral inflow (A) with Valsalva were omitted, given the difficulty in acquiring these measurements in this patient population. **b** Simplified definition of diastolic dysfunction. This is a subset of the parameters presented in the ASE 2009 guidelines. The thresholds used to categorize patients by grade are based on those presented in the ASE guidelines, but have been simplified to include all possible values. *Septal e′* early peak velocity of septal mitral annulus, *LAVI* left atrial volume index, *DT* mitral deceleration time

We measured the diastolic parameters defined in the ASE guidelines: the ratio of early diastolic velocity of mitral inflow (E) to late diastolic velocity of mitral inflow (A), the ratio of E to early diastolic velocity of the septal mitral annulus (e′), left atrial volume index (LAVI), and deceleration time of early diastolic filling (DT). We omitted measurements pertaining to pulmonary venous inflow (Ar-A) or Valsalva maneuver (Valsalva ΔE/A) because pulmonary venous flow images are frequently of limited quality in TTE, and critically ill patients are generally unable to perform a Valsalva maneuver. All measurements of parameters represent the average of measurements from three consecutive cardiac cycles, when available. In rare cases when three consecutive cycles were not captured due to image quality, we used the average of two consecutive cycles. In patients with sinus tachycardia or atrial fibrillation, E and e′ were determined by using previously described methods [2, 21, 22]. We classified diastolic function into four grades (0, 1, 2, and 3) according to the ASE 2009 guidelines (Fig. 1a) [15]. We defined a patient as categorizable if the available measurements unequivocally placed the patient in one of the categories. Conversely, we defined patients as uncategorizable if the available measurements were either discordant or insufficient for placement in a single category.

In addition to assessing diastolic function, we also measured the following: left ventricular ejection fraction (EF), tricuspid annular plane systolic excursion, and tissue Doppler velocity of the septal mitral annulus during ventricular systole (s′).

### Clinical data

We collected demographic information, vital signs, mechanical ventilation parameters, and doses of vasopressors at the time of TTE. We calculated total volume of intravenous fluid administered in the 6 h leading up to and the 6 h after TTE. Central venous pressure was measured at the time of each TTE in patients who had a central venous catheter in place. We calculated the Acute Physiology and Chronic Health Evaluation II (APACHE II) [23] score at the time of ICU admission, ICU-free days to day 28, and the Sequential Organ Failure Assessment (SOFA) score [24] at the time of ICU admission and 72 h after ICU admission. We also determined 28-day all-cause mortality.

### Statistical analysis

We performed multivariable logistic regression for 28-day mortality, with predictors of E, A, E/A, e′, E/e′, DT, and LAVI. Because we anticipated high multicollinearity in these parameters, we also used random forest analysis to determine which of the echocardiographic parameters were most associated with 28-day mortality and ICU-free days to day 28 [25–27]. Using variable importance plots from random forest analysis, we created a simplified definition of diastolic dysfunction based on the e′ and E/e′ (Fig. 1b). We categorized patients according to the ASE 2009 definition and our simplified definition. Differences in 28-day mortality and ICU-free days [28, 29] to day 28 between the groups categorized according to both definitions were compared using Fisher’s exact test or the Kruskal-Wallis test as appropriate. Analyses were performed using Stata release 12 software (StataCorp, College Station, TX, USA) or the R version 3.0.2 statistical software package.

## Results

**Fig. 2:**
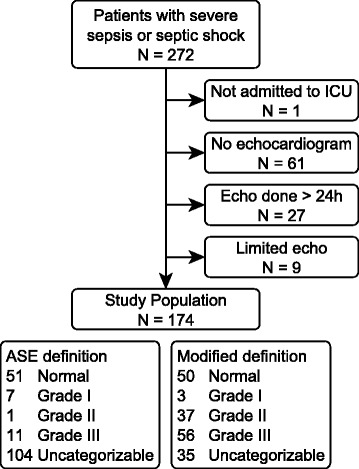
Flowchart representing patient selection. *ICU* intensive care unit, *ASE* American Society of Echocardiography

We studied 167 patients (Fig. 2, Table 1). The median time from meeting enrollment criteria to TTE was 4.3 h. Seven (4 %) of one hundred seventy-four TTE scans were of such poor image quality that no parameters of diastolic function could be measured, and therefore were excluded from the study. Overall 28-day mortality in the cohort was 24 %. The median ICU-free days at 28 days was 25.3 (interquartile range 23.9–26.3). Of the 167 study patients, 47 % had measurements for all five elements of diastolic function from the ASE 2009 guidelines. DT, A, LAVI, e′, and E were unmeasurable in 27 %, 19 %, 16 %, 11 %, and 2 % of patients, respectively. Systolic dysfunction (EF <45 %) was present in 16.2 % of patients. Atrial fibrillation and tachycardia (heart rate >100 beats/minute) were present on 5 % and 44 % of TTE scans, respectively.

**Table 1:** Demographics and clinical findings

Characteristic	Value
Patients, *n*	174
Age, years	64.5 (54–75)
Female sex, %	48.9
APACHE II score	25 (18.25–33)
SOFA score	8 (6–11)
Received vasopressor therapy during admission, %	67.8
Received mechanical ventilation during admission, %	36.8
Overall mortality, %	23.6
Source of infection, %	
Pneumonia	42
Urinary	19.5
Abdominal	11.5
Skin, soft tissue, or joint	11.5
Endocarditis or bacteremia	4.6
CNS	0.6
Multiple	4.2
Uncertain	4.2

*APACHE II* Acute Physiology and Chronic Health Evaluation II, *SOFA* Sequential Organ Failure Assessment, CNS central nervous system

Continuous data are expressed as median (interquartile range)

We performed multivariable logistic regression for 28-day mortality using E, E/A, e′, E/e′, DT, and LAVI as covariates (Table 2). None of these covariates were associated with mortality or ICU-free days in univariate logistic or linear regression, nor were any covariates significantly associated with clinical outcomes after adjusting for SOFA and APACHE II scores. Variable importance plots, generated from random forest analysis of the echocardiographic parameters used in the ASE 2009 definition of diastolic dysfunction, indicated that E velocity, A velocity, E/A ratio, e′ velocity and the E/e′ ratio were most important in predicting 28-day mortality and ICU free days to day 28, while LAVI and DT were unimportant (Additional file 2: Figure S1).

**Table 2:** Multivariable logistic regression for 28-day mortality

	OR	95 % CI	*p* Value
E	0.90	0.84–0.97	0.004
E/A	5.41	1.13–25.95	0.035
e′	2.11	1.00–4.45	0.049
E/e′	1.57	1.07–2.32	0.022
DT	1.00	0.99–1.02	0.641
LAVI	1.03	0.96–1.10	0.477

*Abbreviations: A* late diastolic velocity of mitral inflow, *DT* deceleration time of early diastolic filling, *E* early diastolic velocity of mitral inflow, *e′* early diastolic mitral annular velocity, *E/e′* ratio of early diastolic velocity of mitral inflow to mitral annular velocity, *LAVI* left atrial volume index

The covariates are highly collinear (mean variance inflation factor was 5.3)

On the basis of random forests analysis, we developed a simplified definition of diastolic dysfunction that omitted DT and LAVI (Fig. 1b). We also omitted E/A because (1) in 41 % of patients, the E/A ratio and E/e′ ratio resulted in discordant categorization of diastolic function; (2) A-wave velocities were more often unmeasurable than e′ measurements (19 % vs 11 %) because of either fusion or atrial fibrillation; and (3) review of prior literature so indicated [4–6]. We defined thresholds for grades I–III for the simplified definition based on the ASE 2009 guidelines while adjusting the thresholds to include all values of E/e′ between 8–9 and 12–13.

Using the ASE 2009 definition, we categorized 43 (25.7 %) patients with normal diastolic function and 15 (9.0 %) with diastolic dysfunction (Table 3). We were unable to categorize 109 (65.3 %) patients. By the ASE 2009 definition, 4 patients were uncategorizable because of insufficient data, while 105 had discordant data. In contrast, using the simplified definition, we categorized 50 (29.9 %) patients with normal diastolic function and 96 (57.5 %) with diastolic dysfunction. Twenty-one (12.8 %) patients were considered uncategorizable because of insufficient imaging data. No patients had discordant measurements by the simplified definition. There appeared to be reasonable conservation between the standard and simplified definitions (Additional file 3: Table S2). Among the 62 patients who were categorized under both definitions, only 5 patients (8 %) differed by one grade and none differed by two or more grades.

**Table 3:** Incidence and clinical characteristics of diastolic dysfunction by definition employed

Characteristic	Grade 0	Grade I	Grade II	Grade III	*p* Value
ASE definition (*n* = 58 categorizable patients)					
Patients, *n* (%)	43 (25.7)	8 (5.4)	2 (1.2)	5 (3.0)	<0.01
Age, years	54 (38–70)	71 (64–78)	54 (52–56)	74 (73–82)	0.01
Female sex, %	46.5	50	100	40.0	0.79
Hypertension, %	32.6	25.0	50	100	0.02
Diabetes mellitus, %	25.6	50.0	100	60.0	0.18
Ischemic heart disease, %	7.0	0.0	100	80.0	<0.01
APACHE II score	25 (16–33)	33 (30–35)	28 (11–44)	22 (19–30)	0.64
SOFA score on admission	9 (7–11)	10 (9–12)	9 (7–11)	7 (7–7)	0.22
Percentage on vasopressors during hospitalization	70.0	87.5	50.0	60.0	0.53
BMI, kg/m^2^	27.2 (21.8–36.5)	28.9 (22.1–34.5)	32.3 (28.8–35.9)	28.7 (28.0–36.4)	0.70
E, cm/second	90.1 (77.3–102.5)	57.2 (50.8–93.1)	62.2 (54.1–70.4)	113.6 (112.3–130.5)	<0.01
A, cm/second	74.8 (63.3–92.2)	85.3 (83.9–96.2)	68.6 (59.2–78.0)	34.4 (27.0–41.8)	0.05
e′, cm/second	9.8 (9.0–11.0)	7.0 (5.7–8.6)	7.3 (7.3–7.3)	7.0 (6.1–7.2)	<0.01
E/e′	9.1 (7.6–10.3)	8.1 (7.9–9.4)	9.7 (9.7–9.7)	15.5 (14.6–21.9)	<0.01
DT, milliseconds	180 (158–211)	238 (229–282)	180 (180–180)	142 (121–147)	<0.01
E/A	1.21 (1.04–1.39)	0.67 (0.54–0.87)	0.91 (0.90–0.91)	3.76 (2.69–4.84)	<0.01
LAVI, ml/m^2^	23.7 (15.8–29.6)	55.6 (34.3–59.0)	35.2 (35.2–35.2)	34.7 (34.3–56.5)	<0.01
Ejection fraction	60 (52–70)	75 (72–75)	55 (50–59)	62 (60–65)	0.01
ICU-free days	25.4 (21.3–26.4)	24.8 (19.3–25.4)	25.1 (54.6–25.5)	24.0 (23.2–25.4)	0.42
Mortality, %	27.9	37.5	100	60.0	0.41
IVF administered in 6 h prior to TTE, ml	1231 (600–2000)	714 (141–2005)	633 (633–633)	1000 (1000–1000)	0.75
IVF administered in 6 h after TTE, ml	1200 (575–2085)	500 (105–1486)	1068 (435–1700)	371 (100–1126)	0.40
Simplified definition (*n* = 146 categorizable patients)					
Patients, *n* (%)	50 (34.2)	3 (2.1)	37 (25.3)	56 (38.4)	<0.01
Median age, years	55 (42–70)	86 (70–87)	60 (54–74)	72 (63–77)	<0.01
Female sex, %	46.0	66.7	35.1	64.3	0.03
Hypertension, %	38.0	100	59.5	73.2	<0.01
Diabetes mellitus, %	24.0	100	24.3	57.1	<0.01
Ischemic heart disease, %	10.0	100	13.5	41.1	<0.01
APACHE II score	26 (17–34)	36 (33–37)	25 (16–31)	23 (19–29)	0.19
SOFA score	9 (6–10)	9 (7–11)	9 (7–12)	7 (5–9)	0.07
Percentage on vasopressors during hospitalization	54.0	33.3	37.8	32.1	0.12
BMI, kg/m^2^	27 (22–35)	23 (16–34)	28 (23–32)	28 (24–34)	0.71
E, cm/second	92.1 (80.4)	55.7 (36.7–58.1)	71.1 (58.6–77.7)	101.9 (87.1–117.1)	<0.01
A, cm/second	78.4 (65.8–96.2)	89.3 (67–102.8)	93.5 (76.8–120.1)	93.5 (76.8–120.1)	0.02
e′, cm/second	9.5 (8.6–10.7)	7.0 (6.0–7.4)	6.7 (6.0–7.3)	5.8 (4.7–6.4)	1.00
E/e′	9.3 (7.9–11.4)	7.8 (6.1–7.9)	10.0 (9.6–11.6)	17.7 (14.7–20.6)	<0.01
DT, milliseconds	181 (161–211)	229	208 (191–237)	189 (149–218)	0.05
E/A	1.2 (1.0–1.4)	0.7 (0.4–0.8)	0.8 (0.7–1.0)	1.0 (0.7–1.3)	<0.01
LAVI, ml/m^2^	23.7 (16.2–30.9)	16.6 (166–16.6)	21.0 (16.6–25.1)	25.3 (19.1–31.9)	0.18
Ejection fraction	62 (56–70)	67 (55–75)	61 (50–70)	60 (45–70)	0.52
ICU-free days	25 (22–26)	21 (21–25)	25 (24–26)	25 (24–26)	0.23
Mortality, %	26.0	100	21.6	23.2	0.08
IVF administered in 6 h prior to TTE, ml	1235 (481–2175)	593 (141–1045)	929 (245–2000)	1000 (212–1173)	0.27
IVF administered in 6 h after TTE, ml	1284 (388–2092)	580 (500–5157)	472 (196–1050)	588 (300–1321)	0.05

*Abbreviations: A* late diastolic velocity of mitral inflow, *APACHE II* Acute Physiology and Chronic Health Evaluation II, *ASE* American Society of Echocardiography, *BMI* body mass index, *DT* deceleration time of early diastolic filling, *E* early diastolic velocity of mitral inflow, *e′* early diastolic mitral annular velocity, *E/e′* ratio of early diastolic velocity of mitral inflow to mitral annular velocity, *ICU* intensive care unit, *IVF* intravenous fluid, *LAVI* left atrial volume index, *SOFA* Sequential Organ Failure Assessment, *TTE* transthoracic echocardiography

Continuous data are displayed as medians and interquartile ranges

There were no significant differences in mortality or ICU-free days (regardless of adjustment for SOFA or APACHE II scores) between any of the groups produced using either definition (Table 3). We observed a nonsignificant trend toward higher mortality in patients with grade I diastolic dysfunction (vs all other patients) using the simplified definition (*p* = 0.08), although this finding was based on three patients. We observed significant differences in incidences of diabetes, hypertension, and ischemic heart disease among groups when using the simplified definition, which appeared similar to the differences noted in the ASE 2009 definition (Table 3). In post hoc univariate logistic regression analysis, we found associations between E/e′ and hypertension (OR 1.07, *p* = 0.06), diabetes (OR 1.12, *p* < 0.01), and ischemic heart disease (OR 1.12, *p* < 0.01). Neither definition produced significant differences among grades in regard to APACHE II scores (ASE definition, *p* = 0.13; simplified definition, *p* = 0.19) or SOFA scores on the day of admission to the ICU (ASE definition, *p* = 0.78; simplified definition, *p* = 0.07). Using the simplified definition, patients categorized with normal diastolic function received more intravenous fluid during the subsequent 6 h than those with diastolic dysfunction (median 1284 ml vs 562 ml, *p* = 0.02).

## Discussion

In this large, prospective study, the ASE 2009 definition for assessment of diastolic function did not categorize most patients with severe sepsis and septic shock. Although some classification measures were difficult or impossible to obtain, the primary reason that the ASE 2009 definition failed to categorize most patients was the presence of discordant measurements in many patients. We proposed using a commonly obtainable parameter (E/e′) often used to estimate left atrial pressure [3] as a basis for a simplified definition for diastolic dysfunction that avoids the discordance by omitting unimportant parameters. Using E/e′, we were able to categorize diastolic function in the large majority of patients. Unfortunately, we have no “gold standard” with which to compare our measurements. Physiological measurements to define diastolic function are not feasible in critically ill humans in septic shock. Neither clinical outcomes (i.e., 28-day mortality) nor comparison with the ASE 2009 definition are good tests to determine if the simplified definition is accurate at defining diastolic dysfunction. Although the simplified definition was composed of parameters associated with clinical outcomes, there were no significant differences in 28-day mortality or ICU-free days among the different categories of diastolic dysfunction. A simplified definition of diastolic function avoids discordance, is feasible in patients with sepsis, and offers some information with respect to the distribution of comorbidities associated with diastolic dysfunction (hypertension, ischemic heart disease, diabetes). While further validation of the simplified definition is indicated, the simplified definition offers apparent advantages over definitions derived from non-critically ill populations with no loss of association with clinical outcomes.

Admittedly, the simplest way to reduce discordance is to reduce the number of measurements to be evaluated. In the absence of invasive, intracardiac measurements (often infeasible in these patients), it is difficult to know whether the simplified definition using E/e′ was more accurate than the standard definition. In essence, this study demonstrates that, in patients with sepsis, E/e′ and e′ are the measurements that are commonly available, have no possibility for discordance, and have some association with clinical outcomes. While we found no decrease in association with comorbidities or clinical outcomes with the simplified definition, whether either definition of diastolic dysfunction has prognostic value in this population remains to be seen.

e′ and E/e′ have been identified as predictors of mortality in previous studies [4–6]. Our results support those findings: e′ and E/e′ were associated with 28-day mortality and ICU-free days to day 28. Previous studies have shown that e′ is significantly lower, and E/e′ significantly higher, in nonsurvivors of septic shock [4–6]. Septal e′ and E/e′ have good correlation with left atrial pressure [3] and are also independent predictors of mortality [5, 6]. These studies support the rationale for the simplified definition for evaluating diastolic function in patients with severe sepsis or septic shock. Other studies have shown no association between mortality and e′ or E/e′ [10, 12, 13, 30], but these studies had fewer patients and TTE was typically performed later after the onset of sepsis.

Two other large studies (225 and 262 patients, respectively) in which researchers evaluated diastolic function in the critically ill were limited to patients receiving mechanical ventilation [4, 5]. Our study differs from those studies in two aspects: (1) We performed echocardiography early in the course of sepsis (median time 4.3 h), and (2) we included patients not receiving mechanical ventilation. The inclusion of both patients receiving and patients not receiving mechanical ventilation may increase the generalizability of our results to critical care practice.

Data availability is an important component of why we selected e′ and E/e′ for our simplified definition. A velocity and DT were often unmeasurable in our study (19 % and 27 % of the time, respectively). Atrial fibrillation and tachycardia are common states in severe sepsis or septic shock and may preclude accurate measurement of A or DT. The E and e′ velocities are often still measurable in these patients. It is also uncertain whether other diastolic measurements (LAVI, Ar-A, and Valsalva ΔE/A) are applicable in the critical care setting. High-quality measurement of pulmonary venous flow using TTE may be difficult in critically ill patients [15]. The left atrium, although often measurable, was rarely enlarged. Under the ASE 2009 definition, there is no category for a patient with a normal LAVI and depressed e′. Left atrial enlargement is most likely a manifestation of chronically elevated left ventricular filling pressures and may not reflect acute diastolic dysfunction [31]. In the critical care setting, indices of acute left ventricular filling pressures, such as E/e′, may be more informative to the clinician than a measure of chronic filling pressures, such as the LAVI [3, 32].

We noted no significant differences in clinical outcomes among the groups produced by either definition. It is possible that our study is insufficiently powered to detect differences or that the thresholds we used were inappropriate to discriminate the different grades of diastolic dysfunction in patients with severe sepsis or septic shock. Alternatively, and perhaps more likely, the grade of diastolic dysfunction may not influence outcome in severe sepsis or septic shock. We made no adjustment for severity scores in reporting these assessments, as the APACHE II and SOFA scores were strongly linked to clinical outcomes. Post hoc analyses revealed no association between diastolic dysfunction grade and clinical outcomes after adjusting for disease severity scores in the either definition. Researchers in prior studies reported conflicting data regarding the association between left ventricular dysfunction and clinical outcome [5–7, 11, 14, 30]. Our group previously described increased mortality in patients with grade I diastolic dysfunction, using a definition more in line with the standard ASE definition [1]. The study we report here demonstrated a similar, albeit nonsignificant, trend (*p* = 0.08) based on three patients who died. A larger sample size is needed to draw definite conclusions.

Our study has several limitations. Our definitions of severe sepsis and septic shock [17], although appropriate at the time of the study and used in recent large trials of sepsis [18–20], have been replaced by the Sepsis-3 definitions for sepsis and septic shock [33], meaning that this cohort of patients may not precisely represent patients with sepsis or septic shock described in recent or future publications. The majority of our patients were medical patients, and our findings may not be generalizable to ICUs with different patient populations. We did not use all of the parameters included in the ASE 2009 definition (Ar-A, Valsalva ∆E/A). The lateral annulus was not consistently recorded in many clinical echocardiograms. We therefore used the tissue Doppler measurement of the septal mitral annulus rather than using the average of the septal and lateral measurements. Although this method may overestimate the severity of diastolic dysfunction [34], using septal E/e′ is a reliable method for estimating left atrial pressures [3]. While we recorded receipt of vasopressors and fluid, we did not adjust for these in our analysis.

## Conclusions

Using a simplified version of the ASE 2009 guidelines, we categorized diastolic function in more patients with severe sepsis or septic shock than we did by using the standard ASE 2009 guidelines. While we found some association between specific parameters and clinical outcomes, we found no significant differences in outcomes among any of the groups produced using either definition. Although further study of diastolic function in septic shock is needed, it seems that the simplified definition is more feasible in the critical care setting and offers good agreement with the standard definition.

## Abbreviations

A, late diastolic velocity of mitral inflow; APACHE II, Acute Physiology and Chronic Health Evaluation II; Ar-A, difference in duration flow between atrial reversal of flow and atrial inflow in late diastole; ASE, American Society of Echocardiography; BMI, body mass index; CNS, central nervous system; DT, deceleration time of early diastolic filling; E, early diastolic velocity of mitral inflow; e′, early diastolic mitral annular velocity; E/e′, ratio of early diastolic velocity of mitral inflow to mitral annular velocity; EF, ejection fraction; ICU, intensive care unit; IVF, intravenous fluid; LAVI, left atrial volume index; SOFA, Sequential Organ Failure Assessment; TTE, transthoracic echocardiography

## Additional files

Additional file 1:
**Table S1.** Incidence of diastolic dysfunction reported in prior studies. (DOCX 32 kb) Additional file 2: Variable importance plots. The *vertical bar* is the absolute value of the minimum importance; variables with importance scores greater than this are considered predictive and informative. Mortality, a binary outcome, is measured by change in accuracy of classification. ICU-free days, a continuous outcome, is measured by change in mean square error. (TIF 48 kb) Additional file 3: Table S2. Agreement of simplified definition with the ASE 2009 definition. Insufficient data was defined as insufficient information to classify diastolic function. Discordant was defined as measurements that did not allow for categorization. The simplified definition, by design, does not allow for discordant measurements. (DOCX 25 kb)

## References

[1] Brown SM, Pittman JE, Hirshberg EL, Jones JP, Lanspa MJ, Kuttler KG (2012). Diastolic dysfunction and mortality in early severe sepsis and septic shock: a prospective, observational echocardiography study. Crit Ultrasound J..

[2] Nagueh SF, Kopelen HA, Quinones MA (1996). Assessment of left ventricular filling pressures by Doppler in the presence of atrial fibrillation. Circulation..

[3] Ritzema JL, Richards AM, Crozier IG, Frampton CF, Melton IC, Doughty RN (2011). Serial Doppler echocardiography and tissue Doppler imaging in the detection of elevated directly measured left atrial pressure in ambulant subjects with chronic heart failure. JACC Cardiovasc Imaging..

[4] Landesberg G, Jaffe AS, Gilon D, Levin PD, Goodman S, Abu-Baih A (2014). Troponin elevation in severe sepsis and septic shock: the role of left ventricular diastolic dysfunction and right ventricular dilatation. Crit Care Med..

[5] Landesberg G, Gilon D, Meroz Y, Georgieva M, Levin PD, Goodman S (2012). Diastolic dysfunction and mortality in severe sepsis and septic shock. Eur Heart J..

[6] Sturgess DJ, Marwick TH, Joyce C, Jenkins C, Jones M, Masci P (2010). Prediction of hospital outcome in septic shock: a prospective comparison of tissue Doppler and cardiac biomarkers. Crit Care..

[7] Bouhemad B, Nicolas-Robin A, Arbelot C, Arthaud M, Feger F, Rouby JJ (2008). Isolated and reversible impairment of ventricular relaxation in patients with septic shock. Crit Care Med..

[8] Jafri SM, Lavine S, Field BE, Bahorozian MT, Carlson RW (1990). Left ventricular diastolic function in sepsis. Crit Care Med..

[9] Poelaert J, Declerck C, Vogelaers D, Colardyn F, Visser CA (1997). Left ventricular systolic and diastolic function in septic shock. Intensive Care Med..

[10] Sturgess DJ, Marwick TH, Joyce CJ, Jones M, Venkatesh B (2007). Tissue Doppler in critical illness: a retrospective cohort study. Crit Care..

[11] Munt B, Jue J, Gin K, Fenwick J, Tweeddale M (1998). Diastolic filling in human severe sepsis: an echocardiographic study. Crit Care Med..

[12] McLean AS, Huang SJ, Hyams S, Poh G, Nalos M, Pandit R (2007). Prognostic values of B-type natriuretic peptide in severe sepsis and septic shock. Crit Care Med..

[13] Pulido JN, Afessa B, Masaki M, Yuasa T, Gillespie S, Herasevich V (2012). Clinical spectrum, frequency, and significance of myocardial dysfunction in severe sepsis and septic shock. Mayo Clin Proc..

[14] Micek ST, McEvoy C, McKenzie M, Hampton N, Doherty JA, Kollef MH (2013). Fluid balance and cardiac function in septic shock as predictors of hospital mortality. Crit Care..

[15] Nagueh SF, Appleton CP, Gillebert TC, Marino PN, Oh JK, Smiseth OA (2009). Recommendations for the evaluation of left ventricular diastolic function by echocardiography. J Am Soc Echocardiogr..

[16] Chapman CB, Ewer SM, Kelly AF, Jacobson KM, Leal MA, Rahko PS (2013). Classification of left ventricular diastolic function using American Society of Echocardiography guidelines: agreement among echocardiographers. Echocardiography..

[17] Bone RC, Balk RA, Cerra FB, Dellinger RP, Fein AM, Knaus WA (1992). Definitions for sepsis and organ failure and guidelines for the use of innovative therapies in sepsis. Chest..

[18] Pro CI, Yealy DM, Kellum JA, Huang DT, Barnato AE, Weissfeld LA (2014). A randomized trial of protocol-based care for early septic shock. N Engl J Med..

[19] Mouncey PR, Osborn TM, Power GS, Harrison DA, Sadique MZ, Grieve RD (2015). Trial of early, goal-directed resuscitation for septic shock. N Engl J Med..

[20] ARISE Investigators; ANZICS Clinical Trials Group (2014). Goal-directed resuscitation for patients with early septic shock. N Engl J Med.

[21] Sohn DW, Song JM, Zo JH, Chai IH, Kim HS, Chun HG (1999). Mitral annulus velocity in the evaluation of left ventricular diastolic function in atrial fibrillation. J Am Soc Echocardiogr..

[22] Nagueh SF, Mikati I, Kopelen HA, Middleton KJ, Quinones MA, Zoghbi WA (1998). Doppler estimation of left ventricular filling pressure in sinus tachycardia: a new application of tissue Doppler imaging. Circulation..

[23] Knaus WA, Draper EA, Wagner DP, Zimmerman JE (1985). APACHE II: a severity of disease classification system. Crit Care Med..

[24] Vincent JL, Moreno R, Takala J, Willatts S, De Mendonça A, Bruining H (1996). The SOFA (Sepsis-related Organ Failure Assessment) score to describe organ dysfunction/failure. Intensive Care Med..

[25] Breiman L (2001). Random forests. Mach Learn..

[26] Breiman L (2002). Manual on setting up, using, and understanding random forests v3.1.

[27] Liaw A, Weiener M (2002). Classification and regression by randomForest. R News..

[28] National Heart, Lung, and Blood Institute Acute Respiratory Distress Syndrome (ARDS) Clinical Trials Network (2006). Comparison of two fluid-management strategies in acute lung injury. N Engl J Med.

[29] Grissom CK, Hirshberg EL, Dickerson JB, Brown SM, Lanspa MJ, Liu KD (2015). Fluid management with a simplified conservative protocol for the acute respiratory distress syndrome. Crit Care Med..

[30] Etchecopar-Chevreuil C, Francois B, Clavel M, Pichon N, Gastinne H, Vignon P (2008). Cardiac morphological and functional changes during early septic shock: a transesophageal echocardiographic study. Intensive Care Med..

[31] Pritchett AM, Mahoney DW, Jacobsen SJ, Rodeheffer RJ, Karon BL, Redfield MM (2005). Diastolic dysfunction and left atrial volume: a population-based study. J Am Coll Cardiol..

[32] Nagueh SF, Middleton KJ, Kopelen HA, Zoghbi WA, Quinones MA (1997). Doppler tissue imaging: a noninvasive technique for evaluation of left ventricular relaxation and estimation of filling pressures. J Am Coll Cardiol..

[33] Singer M, Deutschman CS, Seymour CW, Shankar-Hari M, Annane D, Bauer M (2016). The Third International Consensus Definitions for Sepsis and Septic Shock (Sepsis-3). JAMA..

[34] Vignon P, Allot V, Lesage J, Martaille JF, Aldigier JC, Francois B (2007). Diagnosis of left ventricular diastolic dysfunction in the setting of acute changes in loading conditions. Crit Care..

