# Supplementary material for: Application of a simplified definition of diastolic function in severe sepsis and septic shock
Source: Crit Care. 2016 Aug 4;20:243. doi: 10.1186/s13054-016-1421-3 (PMC4973099; doi:10.1186/s13054-016-1421-3)
Supplement: Additional file 1: — Table S1. Incidence of diastolic dysfunction reported in prior studies. (DOCX 32 kb) [file 13054_2016_1421_MOESM1_ESM.docx]

Table e1. Incidence of diastolic dysfunction reported in prior studies.

| Study | N | Patient population | Time and type of echocardiogram | Definition of diastolic dysfunction | Percent with diastolic dysfunction |
| --- | --- | --- | --- | --- | --- |
| Sturgess 2007[^1^](#_ENREF_1) | 94 | General ICU patients | TTE within 5 ± 6 d of ICU admission | e’ < 9.6 cm/s | 67% |
| Etchecopar-Chevreuil 2008[^2^](#_ENREF_2) | 35 | Mechanically ventilated patients with septic shock | TEE within 12 h of ICU admission | e’ < 8.5 cm/s | 20% |
| Bouhemad 2008[^3^](#_ENREF_3) | 54 | Mechanically ventilated, post-operative, septic shock patients | TTE 24 h after onset of septic shock | e’ < 12 cm/s  and  Vp < 45 cm/s | 20% |
| Sturgess 2010[^4^](#_ENREF_4) | 21 | General ICU patients with septic shock | TTE within 72 h of septic shock onset | e’ < 9.6 cm/s  or  E/e’ > 15 | 57% |
| Landesberg 2012[^5^](#_ENREF_5) | 262 | ICU patients with severe sepsis or septic shock | TTE within 72 h of ICU admission | e’ < 8 cm/s | 54.5% |
| Brown 2012[^6^](#_ENREF_6) | 78 | ICU patients with severe sepsis or septic shock | TTE within 0-6 h of severe sepsis or septic shock onset | e’ < 8 cm/s  and  LAVI ≥ 34 ml/m^2^ | 1.4% |
|  |  |  |  | e’ < 8 cm/s | 36.4% |
|  |  |  |  | e’ < 9.6 cm/s  or  E/e’ > 15 | 59.4% |

TTE, transthoracic echocardiogram; TEE, transesophageal echocardiogram; e’, early diastolic peak velocity of septal mitral annulus using tissue Doppler imaging; E, early diastolic mitral inflow using spectral Doppler; LAV, left atrial volume; Vp, propagation velocity

1. Sturgess DJ, Marwick TH, Joyce CJ, Jones M and Venkatesh B. Tissue Doppler in critical illness: a retrospective cohort study. *Crit Care*. 2007;11:R97.

2. Etchecopar-Chevreuil C, Francois B, Clavel M, Pichon N, Gastinne H and Vignon P. Cardiac morphological and functional changes during early septic shock: a transesophageal echocardiographic study. *Intensive Care Med*. 2008;34:250-6.

3. Bouhemad B, Nicolas-Robin A, Arbelot C, Arthaud M, Feger F and Rouby JJ. Isolated and reversible impairment of ventricular relaxation in patients with septic shock. *Crit Care Med*. 2008;36:766-74.

4. Sturgess DJ, Marwick TH, Joyce C, Jenkins C, Jones M, Masci P, Stewart D and Venkatesh B. Prediction of hospital outcome in septic shock: a prospective comparison of tissue Doppler and cardiac biomarkers. *Crit Care*. 2010;14:R44.

5. Landesberg G, Gilon D, Meroz Y, Georgieva M, Levin PD, Goodman S, Avidan A, Beeri R, Weissman C, Jaffe AS and Sprung CL. Diastolic dysfunction and mortality in severe sepsis and septic shock. *Eur Heart J*. 2012;33:895-903.

6. Brown SM, Pittman JE, Hirshberg EL, Jones JP, Lanspa MJ, Kuttler KG, Litwin SE and Grissom CK. Diastolic dysfunction and mortality in early severe sepsis and septic shock: a prospective, observational echocardiography study. *Critical ultrasound journal*. 2012;4:8.
