# Supplementary figures and images for: Application of a simplified definition of diastolic function in severe sepsis and septic shock
Source: Crit Care. 2016 Aug 4;20:243. doi: 10.1186/s13054-016-1421-3 (PMC4973099; doi:10.1186/s13054-016-1421-3)

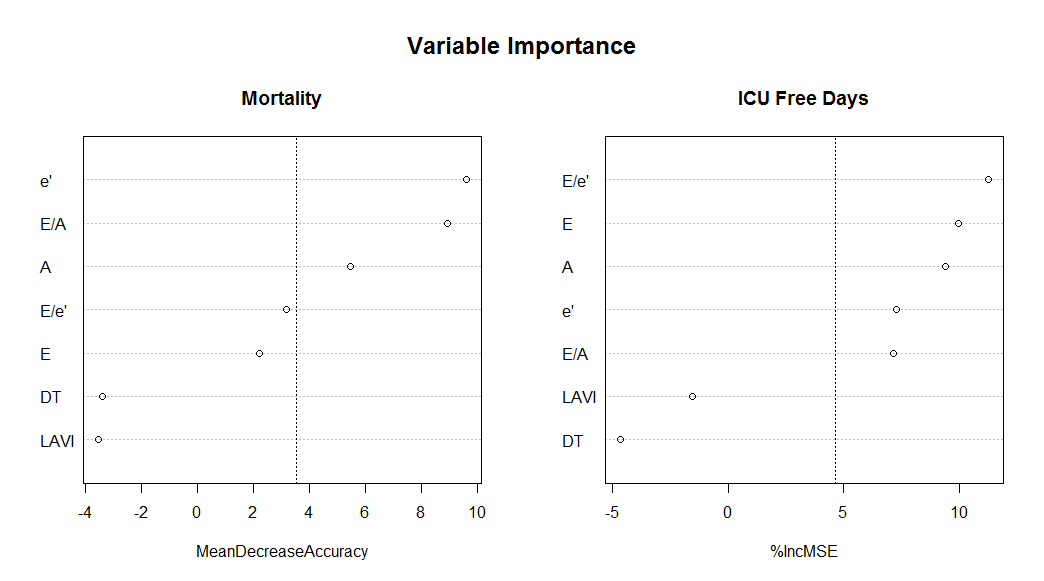

Supplement: Additional file 2: — Variable importance plots. The vertical bar is the absolute value of the minimum importance; variables with importance scores greater than this are considered predictive and informative. Mortality, a binary outcome, is measured by change in accuracy of classification. ICU-free days, a continuous outcome, is measured by change in mean square error. (TIF 48 kb) [file 13054_2016_1421_MOESM2_ESM.tif]
