# Supplementary material for: Application of a simplified definition of diastolic function in severe sepsis and septic shock
Source: Crit Care. 2016 Aug 4;20:243. doi: 10.1186/s13054-016-1421-3 (PMC4973099; doi:10.1186/s13054-016-1421-3)
Supplement: Additional file 3: Table S2. — Agreement of simplified definition with the ASE 2009 definition. Insufficient data was defined as insufficient information to classify diastolic function. Discordant was defined as measurements that did not allow for categorization. The simplified definition, by design, does not allow for discordant measurements. (DOCX 25 kb) [file 13054_2016_1421_MOESM3_ESM.docx]

Table e2. Agreement of simplified definition with the ASE 2009 definition. Insufficient Data: insufficient information to classify diastolic function. Discordant: measurements that do not allow for categorization. The simplified definition, by design, does not allow for discordant measurements.

|  | Normal | Grade 1 | Grade 2 | Grade 3 | Insufficient data |
| --- | --- | --- | --- | --- | --- |
| ASE definition: |  |  |  |  |  |
| Normal | 39 | 0 | 0 | 0 | 4 |
| Grade 1 | 2 | 2 | 3 | 0 | 1 |
| Grade 2 | 0 | 0 | 1 | 0 | 1 |
| Grade 3 | 0 | 0 | 0 | 5 | 0 |
| Insufficient data | 0 | 0 | 0 | 1 | 3 |
| Discordant | 9 | 1 | 33 | 50 | 12 |
